# 10′-Chloro-3′,4′-dihydro-2′*H*-spiro­[cyclo­propane-1,7′(6′*H*)-pyrimido[2,1-*a*]isoquinolin]-6′-one

**DOI:** 10.1107/S1600536812044261

**Published:** 2012-10-31

**Authors:** Kensuke Okuda, Takashi Hirota, Yuta Nishina, Hiroyuki Ishida

**Affiliations:** a Laboratory of Medicinal and Pharmaceutical Chemistry, Gifu Pharmaceutical University, Gifu 501-1196, Japan; b Laboratory of Pharmaceutical Chemistry, Faculty of Pharmaceutical Sciences, Okayama University, Okayama 700-8530, Japan; c Research Core for Interdisciplinary Sciences, Okayama University, Okayama 700-8530, Japan; d Department of Chemistry, Faculty of Science, Okayama University, Okayama 700-8530, Japan

## Abstract

In the title compound, C_14_H_13_ClN_2_O, the fused hydro­pyrimidine ring adopts an envelope conformation with one of the methyl­ene C atoms at the flap. The three-membered ring is approximately perpendicular to the attached isoquinoline ring system, with a dihedral angle of 89.44 (11)°. In the crystal, mol­ecules are linked by a weak C—H⋯π inter­action, forming a helical chain along the *c* axis.

## Related literature

For recent reports on the development of complex heterocyclic skeletons for potential pharmaceutics in one step using the Truce–Smiles rearrangement, see: Okuda *et al.* (2010 ▶, 2011 ▶). For the synthesis of the title compound, see: Okuda *et al.* (2012 ▶).
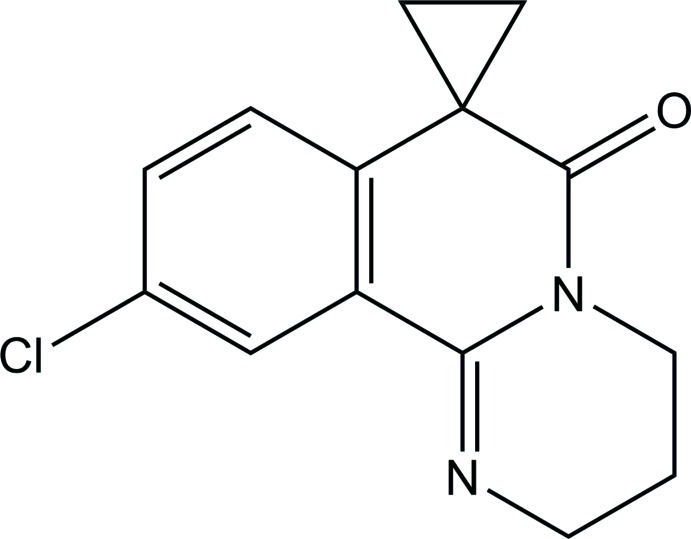


## Experimental

### 

#### Crystal data

C_14_H_13_ClN_2_O
*M* *_r_* = 260.72
Orthorhombic, 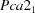

*a* = 8.8746 (5) Å
*b* = 13.3273 (7) Å
*c* = 9.8331 (6) Å
*V* = 1163.01 (11) Å^3^
*Z* = 4
Mo *K*α radiation
μ = 0.32 mm^−1^
*T* = 180 K
0.25 × 0.21 × 0.03 mm

#### Data collection

Rigaku R-AXIS RAPIDII diffractometer
Absorption correction: numerical (*NUMABS*; Higashi, 1999 ▶) *T*_min_ = 0.931, *T*_max_ = 0.991
17373 measured reflections
3359 independent reflections
3095 reflections with *I* > 2σ(*I*)
*R*_int_ = 0.027

#### Refinement

*R*[*F* ^2^ > 2σ(*F* ^2^)] = 0.031
*wR*(*F* ^2^) = 0.079
*S* = 1.05
3359 reflections
163 parameters
1 restraint
H-atom parameters constrained
Δρ_max_ = 0.32 e Å^−3^
Δρ_min_ = −0.15 e Å^−3^
Absolute structure: Flack (1983 ▶), 1571 Friedel pairs
Flack parameter: 0.01 (5)

### 

Data collection: *PROCESS-AUTO* (Rigaku/MSC, 2004 ▶); cell refinement: *PROCESS-AUTO*; data reduction: *CrystalStructure* (Rigaku/MSC, 2004 ▶); program(s) used to solve structure: *SHELXS97* (Sheldrick, 2008 ▶); program(s) used to refine structure: *SHELXL97* (Sheldrick, 2008 ▶); molecular graphics: *ORTEP-3* (Farrugia, 1997 ▶); software used to prepare material for publication: *SHELXL97* and *PLATON* (Spek, 2009 ▶).

## Supplementary Material

Crystal structure: contains datablock(s) global, I. DOI: 10.1107/S1600536812044261/ff2085sup1.cif

Structure factors: contains datablock(s) I. DOI: 10.1107/S1600536812044261/ff2085Isup2.hkl

Additional supplementary materials:  crystallographic information; 3D view; checkCIF report

## Figures and Tables

**Table 1:** Hydrogen-bond geometry (Å, °) *Cg*1 is the centroid of the C1/N1/C2–C4/C9 ring.

*D*—H⋯*A*	*D*—H	H⋯*A*	*D*⋯*A*	*D*—H⋯*A*
C12—H12*B*⋯*Cg*1^i^	0.99	2.72	3.6000 (15)	148

Symmetry code: (i) 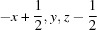.

**Fig. 1.**
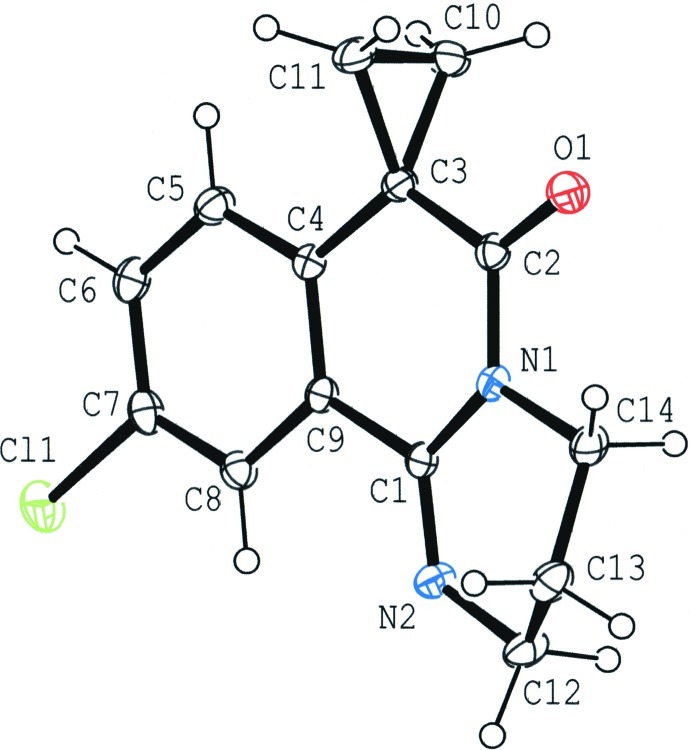
The molecular structure of the title compound, with the atom-labeling. Displacement ellipsoids of non-H atoms are drawn at the 50% probability level.

**Fig. 2.**
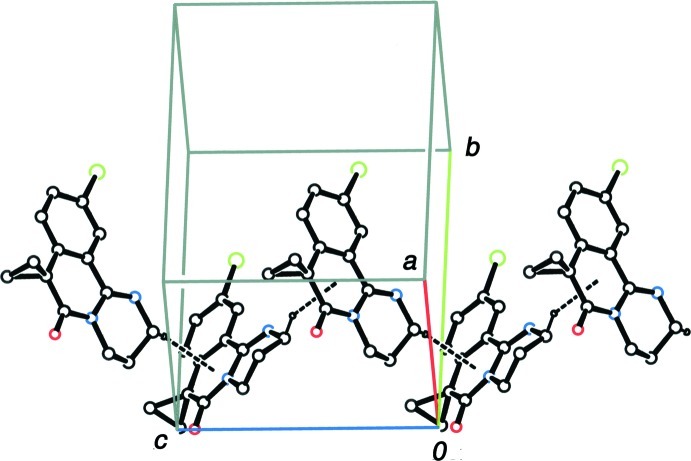
A packing diagram of the title compound, showing a chain structure formed by C—H···π interactions (dashed lines).

## supplementary crystallographic information

### Comment

As an extension of our work to develop complex heterocyclic skeletons as leads for potential pharmaceutical agents (Okuda *et al.*, 2011), we found that tricyclic 5-amino-1,2-dihydrofuro[2,3-*c*]isoquinolines (Okuda *et al.*, 2010), easily accessible by a one step base-induced Truce-Smiles rearrangement of 2-(3-cyanopropoxy)benzonitriles, showed bronchodilator activity (unpublished results). In the pursuit of more potent analogs, we have explored preparation of additional new ring-fused tetracyclic heterocycles. Herein we report that reaction of 5-amino-7-chloro-1,2-dihydrofuro[2,3-*c*]isoquinoline with 1,3-dibromopropane in the presence of calcium oxide afforded the title compound (Okuda *et al.*, 2012) *via* rearrangement instead of the anticipated 10-chloro-2,3,6,7-tetrahydrofuro[2,3-*c*]imidazo[2,1-*a*]isoquinoline.

In the title compound, the fused hydropyrimidine N1/C1/N2/C12–C14 ring adopts an envelope conformation with atom C13 at the flap. The isoquinoline C1/N1/C2–C9 ring system is planar with an *r.m.s.* deviation of 0.044 (1) Å. The three-membered C3/C10/C11 ring is approximately perpendicular to the attached isoquinoline ring system with a dihedral angle of 89.44 (11)°. In the crystal, molecules are linked by a weak C—H···π interaction (Table 1), forming a helical chain along the *c* axis.

### Experimental

The detailed experimental procedure for the synthesis of 10'-chloro-3',4'-dihydro-2'*H*-spiro[cyclopropane-1,7'(6'*H*)-pyrimido[2,1-*a*]isoquinolin]-6'-one (m.p. 424–425 K from *n*-hexane) from 5-amino-7-chloro-1,2-dihydrofuro[2,3-*c*]isoquinoline was described in our previous paper (Okuda *et al.*, 2012). Single crystals suitable for X-ray diffraction were obtained from an acetonitrile/water solution. The title compound was dissolved in hot acetonitrile, then water was added dropwise until the solution became turbid. Slow evaporation at room temperature gave the colourless crystals

### Refinement

H atoms were located in a difference Fourier map and then were positioned geometrically (C—H = 0.95 or 0.99 Å) and refined as riding, with *U*_iso_(H) = 1.2*U*_eq_(C).

### Crystal data

**Table:**

C_14_H_13_ClN_2_O	*F*(000) = 544.00
*M**_r_* = 260.72	*D*_x_ = 1.489 Mg m^−^^3^
Orthorhombic, *P**c**a*2_1_	Mo *K*α radiation, λ = 0.71075 Å
Hall symbol: P 2c -2ac	Cell parameters from 15021 reflections
*a* = 8.8746 (5) Å	θ = 3.1–30.0°
*b* = 13.3273 (7) Å	µ = 0.32 mm^−^^1^
*c* = 9.8331 (6) Å	*T* = 180 K
*V* = 1163.01 (11) Å^3^	Platelet, colourless
*Z* = 4	0.25 × 0.21 × 0.03 mm

### Data collection

**Table:**

Rigaku R-AXIS RAPIDII diffractometer	3095 reflections with *I* > 2σ(*I*)
Detector resolution: 10.00 pixels mm^-1^	*R*_int_ = 0.027
ω scans	θ_max_ = 30.0°
Absorption correction: numerical (*NUMABS*; Higashi, 1999)	*h* = −12→12
*T*_min_ = 0.931, *T*_max_ = 0.991	*k* = −18→18
17373 measured reflections	*l* = −13→13
3359 independent reflections	

### Refinement

**Table:**

Refinement on *F*^2^	Secondary atom site location: difference Fourier map
Least-squares matrix: full	Hydrogen site location: inferred from neighbouring sites
*R*[*F*^2^ > 2σ(*F*^2^)] = 0.031	H-atom parameters constrained
*wR*(*F*^2^) = 0.079	*w* = 1/[σ^2^(*F*_o_^2^) + (0.0483*P*)^2^ + 0.1225*P*] where *P* = (*F*_o_^2^ + 2*F*_c_^2^)/3
*S* = 1.05	(Δ/σ)_max_ = 0.001
3359 reflections	Δρ_max_ = 0.32 e Å^−^^3^
163 parameters	Δρ_min_ = −0.15 e Å^−^^3^
1 restraint	Absolute structure: Flack (1983), 1571 Friedel pairs
Primary atom site location: structure-invariant direct methods	Flack parameter: 0.01 (5)

### Special details

**Table:**

Geometry. All e.s.d.'s (except the e.s.d. in the dihedral angle between two l.s. planes) are estimated using the full covariance matrix. The cell e.s.d.'s are taken into account individually in the estimation of e.s.d.'s in distances, angles and torsion angles; correlations between e.s.d.'s in cell parameters are only used when they are defined by crystal symmetry. An approximate (isotropic) treatment of cell e.s.d.'s is used for estimating e.s.d.'s involving l.s. planes.
Refinement. Refinement of *F*^2^ against ALL reflections. The weighted *R*-factor *wR* and goodness of fit *S* are based on *F*^2^, conventional *R*-factors *R* are based on *F*, with *F* set to zero for negative *F*^2^. The threshold expression of *F*^2^ > σ(*F*^2^) is used only for calculating *R*-factors(gt) *etc*. and is not relevant to the choice of reflections for refinement. *R*-factors based on *F*^2^ are statistically about twice as large as those based on *F*, and *R*- factors based on ALL data will be even larger.

### Fractional atomic coordinates and isotropic or equivalent isotropic displacement parameters (Å^2^)

**Table:**

	*x*	*y*	*z*	*U*_iso_*/*U*_eq_	
Cl1	0.55266 (4)	0.63700 (2)	0.30542 (5)	0.03531 (10)	
O1	0.57503 (12)	0.04186 (7)	0.43848 (12)	0.0307 (2)	
N1	0.45976 (11)	0.15982 (7)	0.31067 (13)	0.01989 (19)	
N2	0.36794 (13)	0.28236 (8)	0.15502 (12)	0.0249 (2)	
C1	0.44659 (13)	0.25802 (10)	0.25827 (13)	0.0189 (2)	
C2	0.56200 (15)	0.13087 (10)	0.40789 (14)	0.0211 (2)	
C3	0.65462 (14)	0.20982 (9)	0.47518 (13)	0.0205 (2)	
C4	0.63116 (14)	0.31618 (9)	0.43507 (12)	0.0191 (2)	
C5	0.70816 (15)	0.39510 (11)	0.49866 (14)	0.0259 (3)	
H5	0.7774	0.3806	0.5697	0.031*	
C6	0.68510 (16)	0.49385 (10)	0.45973 (15)	0.0260 (3)	
H6	0.7372	0.5470	0.5038	0.031*	
C7	0.58418 (16)	0.51363 (10)	0.35496 (15)	0.0242 (3)	
C8	0.50749 (14)	0.43794 (9)	0.28990 (14)	0.0226 (2)	
H8	0.4391	0.4531	0.2185	0.027*	
C9	0.53128 (13)	0.33791 (9)	0.33006 (13)	0.0187 (2)	
C10	0.81354 (17)	0.17547 (12)	0.51350 (16)	0.0301 (3)	
H10A	0.8443	0.1069	0.4865	0.036*	
H10B	0.8952	0.2260	0.5108	0.036*	
C11	0.69779 (19)	0.18535 (11)	0.62195 (15)	0.0293 (3)	
H11A	0.7079	0.2420	0.6864	0.035*	
H11B	0.6570	0.1229	0.6621	0.035*	
C12	0.27594 (16)	0.20671 (11)	0.08694 (15)	0.0297 (3)	
H12A	0.3328	0.1794	0.0086	0.036*	
H12B	0.1836	0.2390	0.0513	0.036*	
C13	0.23159 (15)	0.12115 (11)	0.17992 (16)	0.0289 (3)	
H13A	0.1610	0.1456	0.2505	0.035*	
H13B	0.1802	0.0681	0.1268	0.035*	
C14	0.37139 (16)	0.07873 (10)	0.24647 (16)	0.0267 (3)	
H14A	0.3421	0.0288	0.3162	0.032*	
H14B	0.4338	0.0443	0.1773	0.032*	

### Atomic displacement parameters (Å^2^)

**Table:**

	*U*^11^	*U*^22^	*U*^33^	*U*^12^	*U*^13^	*U*^23^
Cl1	0.04450 (18)	0.01793 (13)	0.0435 (2)	−0.00198 (13)	0.00151 (18)	0.00384 (15)
O1	0.0391 (5)	0.0195 (5)	0.0334 (6)	−0.0010 (4)	−0.0080 (5)	0.0010 (4)
N1	0.0222 (4)	0.0189 (4)	0.0186 (5)	−0.0024 (3)	−0.0003 (4)	−0.0022 (5)
N2	0.0257 (5)	0.0269 (5)	0.0221 (5)	−0.0025 (4)	−0.0051 (4)	0.0002 (4)
C1	0.0193 (5)	0.0197 (5)	0.0177 (5)	0.0001 (4)	0.0020 (4)	−0.0015 (4)
C2	0.0244 (5)	0.0197 (6)	0.0190 (6)	−0.0007 (4)	0.0012 (4)	0.0000 (4)
C3	0.0227 (5)	0.0201 (5)	0.0187 (5)	−0.0006 (4)	−0.0026 (4)	−0.0005 (4)
C4	0.0205 (5)	0.0185 (5)	0.0184 (5)	−0.0011 (4)	0.0005 (4)	−0.0012 (4)
C5	0.0280 (6)	0.0252 (6)	0.0245 (6)	−0.0031 (5)	−0.0051 (5)	−0.0013 (5)
C6	0.0295 (6)	0.0215 (6)	0.0270 (7)	−0.0065 (5)	0.0022 (5)	−0.0046 (5)
C7	0.0279 (6)	0.0176 (5)	0.0272 (6)	−0.0009 (5)	0.0069 (5)	0.0009 (5)
C8	0.0239 (5)	0.0209 (5)	0.0231 (6)	0.0005 (4)	0.0005 (5)	0.0008 (5)
C9	0.0193 (5)	0.0194 (5)	0.0174 (6)	−0.0005 (4)	0.0020 (4)	−0.0013 (4)
C10	0.0273 (7)	0.0278 (7)	0.0351 (8)	0.0024 (6)	−0.0106 (6)	−0.0001 (6)
C11	0.0407 (8)	0.0257 (6)	0.0214 (6)	−0.0004 (6)	−0.0089 (6)	0.0032 (5)
C12	0.0302 (7)	0.0331 (7)	0.0259 (7)	−0.0057 (6)	−0.0092 (6)	−0.0028 (5)
C13	0.0256 (6)	0.0330 (7)	0.0281 (7)	−0.0072 (5)	−0.0040 (5)	−0.0051 (6)
C14	0.0321 (6)	0.0200 (6)	0.0282 (6)	−0.0051 (5)	−0.0052 (5)	−0.0039 (5)

### Geometric parameters (Å, º)

**Table:**

Cl1—C7	1.7376 (14)	C6—H6	0.9500
O1—C2	1.2292 (16)	C7—C8	1.3748 (19)
N1—C2	1.3733 (17)	C8—C9	1.4063 (17)
N1—C1	1.4113 (16)	C8—H8	0.9500
N1—C14	1.4769 (16)	C10—C11	1.486 (2)
N2—C1	1.2740 (17)	C10—H10A	0.9900
N2—C12	1.4599 (17)	C10—H10B	0.9900
C1—C9	1.4822 (17)	C11—H11A	0.9900
C2—C3	1.4902 (18)	C11—H11B	0.9900
C3—C4	1.4859 (17)	C12—C13	1.514 (2)
C3—C11	1.5284 (19)	C12—H12A	0.9900
C3—C10	1.5299 (19)	C12—H12B	0.9900
C4—C9	1.3913 (17)	C13—C14	1.512 (2)
C4—C5	1.4015 (18)	C13—H13A	0.9900
C5—C6	1.3859 (19)	C13—H13B	0.9900
C5—H5	0.9500	C14—H14A	0.9900
C6—C7	1.390 (2)	C14—H14B	0.9900
			
C2—N1—C1	124.71 (10)	C4—C9—C1	121.81 (11)
C2—N1—C14	116.28 (10)	C8—C9—C1	118.10 (11)
C1—N1—C14	118.59 (11)	C11—C10—C3	60.87 (9)
C1—N2—C12	119.73 (12)	C11—C10—H10A	117.7
N2—C1—N1	124.92 (12)	C3—C10—H10A	117.7
N2—C1—C9	118.32 (12)	C11—C10—H10B	117.7
N1—C1—C9	116.76 (11)	C3—C10—H10B	117.7
O1—C2—N1	120.24 (12)	H10A—C10—H10B	114.8
O1—C2—C3	121.39 (12)	C10—C11—C3	60.97 (9)
N1—C2—C3	118.37 (11)	C10—C11—H11A	117.7
C4—C3—C2	118.58 (11)	C3—C11—H11A	117.7
C4—C3—C11	119.30 (11)	C10—C11—H11B	117.7
C2—C3—C11	114.01 (11)	C3—C11—H11B	117.7
C4—C3—C10	118.68 (11)	H11A—C11—H11B	114.8
C2—C3—C10	113.99 (11)	N2—C12—C13	112.88 (12)
C11—C3—C10	58.16 (9)	N2—C12—H12A	109.0
C9—C4—C5	119.05 (11)	C13—C12—H12A	109.0
C9—C4—C3	119.00 (11)	N2—C12—H12B	109.0
C5—C4—C3	121.94 (11)	C13—C12—H12B	109.0
C6—C5—C4	121.16 (12)	H12A—C12—H12B	107.8
C6—C5—H5	119.4	C14—C13—C12	109.24 (11)
C4—C5—H5	119.4	C14—C13—H13A	109.8
C5—C6—C7	118.68 (12)	C12—C13—H13A	109.8
C5—C6—H6	120.7	C14—C13—H13B	109.8
C7—C6—H6	120.7	C12—C13—H13B	109.8
C8—C7—C6	121.64 (12)	H13A—C13—H13B	108.3
C8—C7—Cl1	118.96 (11)	N1—C14—C13	110.31 (11)
C6—C7—Cl1	119.40 (10)	N1—C14—H14A	109.6
C7—C8—C9	119.38 (12)	C13—C14—H14A	109.6
C7—C8—H8	120.3	N1—C14—H14B	109.6
C9—C8—H8	120.3	C13—C14—H14B	109.6
C4—C9—C8	120.09 (11)	H14A—C14—H14B	108.1
			
C12—N2—C1—N1	3.66 (19)	C4—C5—C6—C7	−0.4 (2)
C12—N2—C1—C9	−176.29 (11)	C5—C6—C7—C8	0.0 (2)
C2—N1—C1—N2	169.27 (13)	C5—C6—C7—Cl1	179.55 (11)
C14—N1—C1—N2	−2.93 (19)	C6—C7—C8—C9	0.1 (2)
C2—N1—C1—C9	−10.78 (18)	Cl1—C7—C8—C9	−179.46 (10)
C14—N1—C1—C9	177.02 (11)	C5—C4—C9—C8	−0.63 (18)
C1—N1—C2—O1	−173.21 (13)	C3—C4—C9—C8	−179.79 (11)
C14—N1—C2—O1	−0.84 (19)	C5—C4—C9—C1	179.82 (11)
C1—N1—C2—C3	7.29 (19)	C3—C4—C9—C1	0.66 (17)
C14—N1—C2—C3	179.66 (12)	C7—C8—C9—C4	0.24 (18)
O1—C2—C3—C4	−178.91 (12)	C7—C8—C9—C1	179.81 (11)
N1—C2—C3—C4	0.58 (18)	N2—C1—C9—C4	−173.56 (12)
O1—C2—C3—C11	−30.41 (18)	N1—C1—C9—C4	6.49 (17)
N1—C2—C3—C11	149.08 (13)	N2—C1—C9—C8	6.88 (17)
O1—C2—C3—C10	33.87 (19)	N1—C1—C9—C8	−173.07 (11)
N1—C2—C3—C10	−146.63 (13)	C4—C3—C10—C11	108.47 (14)
C2—C3—C4—C9	−4.26 (17)	C2—C3—C10—C11	−104.35 (13)
C11—C3—C4—C9	−151.08 (12)	C4—C3—C11—C10	−107.42 (14)
C10—C3—C4—C9	141.41 (12)	C2—C3—C11—C10	104.32 (13)
C2—C3—C4—C5	176.60 (12)	C1—N2—C12—C13	25.27 (18)
C11—C3—C4—C5	29.78 (18)	N2—C12—C13—C14	−52.69 (16)
C10—C3—C4—C5	−37.73 (18)	C2—N1—C14—C13	160.89 (12)
C9—C4—C5—C6	0.73 (19)	C1—N1—C14—C13	−26.25 (17)
C3—C4—C5—C6	179.87 (13)	C12—C13—C14—N1	51.96 (16)

### Hydrogen-bond geometry (Å, º)

*Cg*1 is the centroid of the C1/N1/C2–C4/C9 ring.

**Table:**

*D*—H···*A*	*D*—H	H···*A*	*D*···*A*	*D*—H···*A*
C12—H12*B*···*Cg*1^i^	0.99	2.72	3.6000 (15)	148

Symmetry code: (i) −*x*+1/2, *y*, *z*−1/2.
